# Traumatic Periprosthetic Acetabular Fracture Treated with One-Stage Exchange and Bone Reconstruction Using a Synthetic Bone Graft Substitute

**DOI:** 10.1155/2016/4160128

**Published:** 2016-06-30

**Authors:** Jan Svacina

**Affiliations:** Department of Orthopaedic Surgery, Bodden-Kliniken Ribnitz-Damgarten GmbH, 18311 Ribnitz-Damgarten, Germany

## Abstract

A case of a traumatic periprosthetic acetabular fracture in an elderly patient, which was treated by one-stage hip exchange with implantation of an antiprotrusio cage and reconstruction of the acetabular bone loss with an injectable calcium sulphate/hydroxyapatite bone graft substitute, is reported. The paste-like bone graft substitute was injected through the holes of the antiprotrusio cage. After a setting time of 15 minutes, a low-profile cup was cemented onto the cage using polymethylmethacrylate and a new stem was inserted. The patient was encouraged to ambulate three days postoperatively weight-bearing as tolerated. At the one-year follow-up visit the patient was ambulatory and full weight-bearing without any walking aids. The follow-up radiographs demonstrated stable position and articulation of the revision hip arthroplasty with no signs of loosening of the antiprotrusio cage. However, the most interesting finding was that the bone graft substitute had remodelled to a great extent into bone. This calcium sulphate/hydroxyapatite composite shows high osteoconductive potential and can be used to regenerate bone stock in revision arthroplasty.

## 1. Introduction

Periprosthetic acetabular fractures are rare injuries, but their incidence is rising due to increased prevalence of total hip arthroplasty [1]. They can occur intraoperatively during the insertion of the acetabular component [2] or postoperatively as a result of osteolytic pelvic lesions [3] or periprosthetic insufficiency fractures [4]. Only a few reports about traumatic periprosthetic fractures of the acetabulum and their management are available in the literature [3, 5].

We present the case of a traumatic periprosthetic acetabular fracture (central protrusion fracture) treated by one-stage exchange using a Burch-Schneider antiprotrusio cage and bone reconstruction with a calcium sulphate/hydroxyapatite bone graft substitute.

## 2. Case Presentation

An 84-year-old male was admitted to hospital after a fall, complaining of pain at his left hip and inability to walk. Seven weeks before a cementless total hip arthroplasty (T.O.P. cup, polyethylene inlay, ceramic head 32 M, Link, Hamburg, Germany, and AL 2000 shaft, size 8, Speetec Implantate GmbH, Langelsheim, Germany) had been implanted due to severe osteoarthritis of the left hip. No complications had occurred after surgery or during rehabilitation.

**Figure 1:**
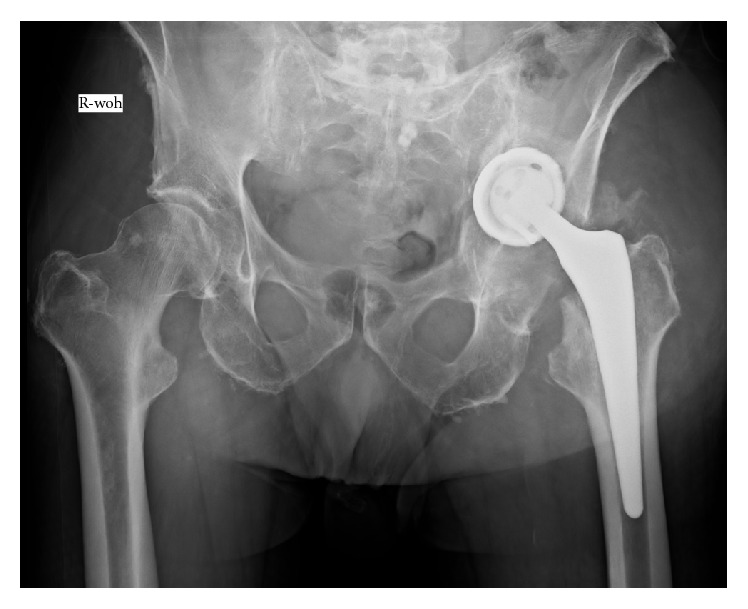
Radiograph of pelvis (deep) after fall: periprosthetic acetabular fracture (type IIIa acetabular defect according to Paprosky et al. [20]).

The clinical and X-ray examinations revealed a periprosthetic acetabular fracture with dislocation of the loosened cup into the small pelvis (Figure 1). The patient was hemodynamically stable. The fracture was assessed as central protrusion fracture and the fracture type classified as IV.6 B3 (UCS classification for periprosthetic fractures) [6]. According to the classification and recommendation of Peterson II and Lewallen [7], for this type II fracture (loosening of the THA cup-component) we planned a surgical revision with exchange of the loosened cup, additional fracture augmentation, and bone reconstruction with a synthetic bone graft substitute. Informed consent of the patient was obtained.

**Figure 2:**
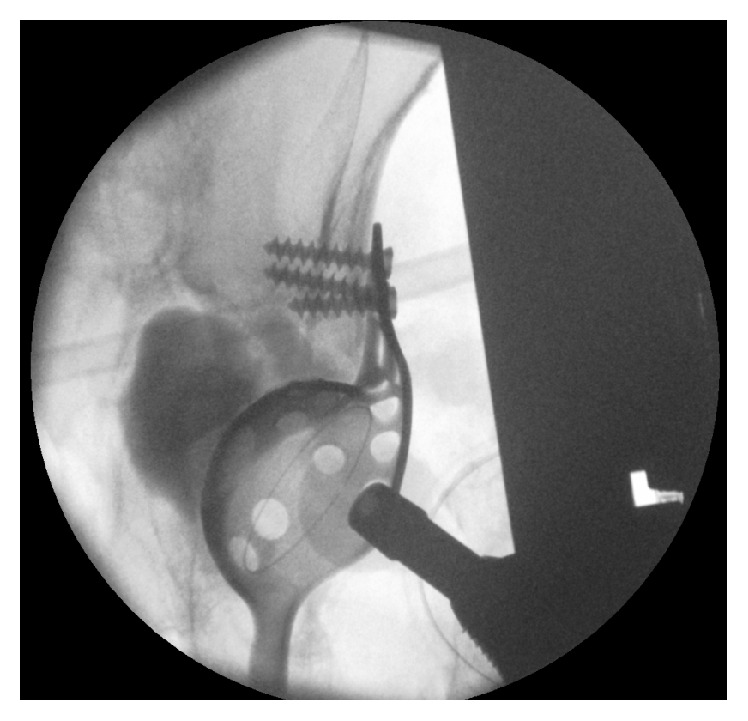
Fluoroscopy during surgery: acetabular fracture reduced, Burch-Schneider antiprotrusio cage implanted, and CERAMENT|BVF injected behind the cage.

**Figure 3:**
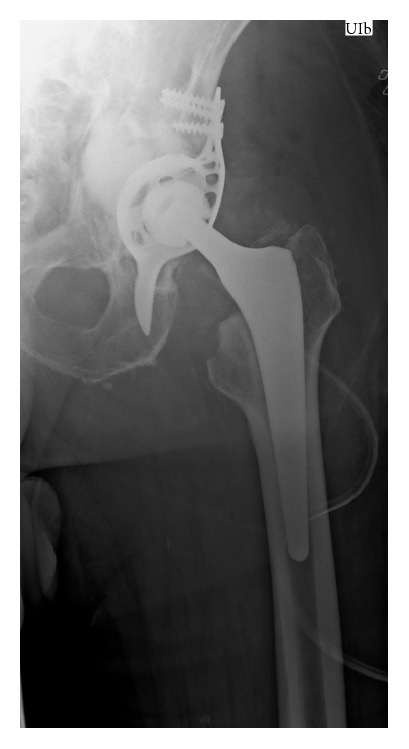
Radiograph of left hip a.p. (supine) on the second day postoperatively.

Surgery was performed four days after admission. The patient was placed on a radiolucent table in supine position. The anterior-lateral approach of the initial surgery was used. The loosened cup and the femoral stem were removed. The fracture was reduced and a Burch-Schneider antiprotrusio cage (size 50, Zimmer, Freiburg im Breisgau, Germany) implanted. The significant acetabular bone loss was filled with 18 mL CERAMENT*™*|BONE VOID FILLER (Bonesupport, Lund, Sweden) under fluoroscopic control (Figure 2). This paste-like calcium sulphate/hydroxyapatite bone graft substitute was injected through the holes of the Burch-Schneider cage. After a setting time of 15 minutes, a low-profile cup (size 48, Longevity, Durasul PE, with a ceramic head 32 M, Zimmer, Freiburg im Breisgau, Germany) was cemented using polymethylmethacrylate (PMMA). Finally, a new stem (AL 2000, size 9, Speetec Implantate GmbH, Langelsheim, Germany) was inserted in a press-fit technique. The patient received a total of five erythrocyte concentrates, two intraoperatively and three postoperatively. The postoperative course was event-free. X-ray confirmed correct position of the antiprotrusio cage and the revision THA (Figure 3). The incision healed* per primam intentionem *without prolonged wound drainage. Diclofenac 75 mg (Ratiopharm GmbH, Ulm, Germany) orally once a day was used as prevention of heterotopic ossifications. The patient was encouraged to ambulate three days postoperatively weight-bearing as tolerated, first with the support of a walking frame and then with a rollator-walker. At postoperative day 17 the patient was discharged and a week later rehabilitation started in an in-patient setting.

### 2.1. Follow-Up at One Year

At the one-year follow-up visit the patient was ambulatory and full weight-bearing without any walking aids. Extension/flexion of the left hip was 0°-0°-80° and external/internal rotation 20°-0°-10°.

**Figure 4:**
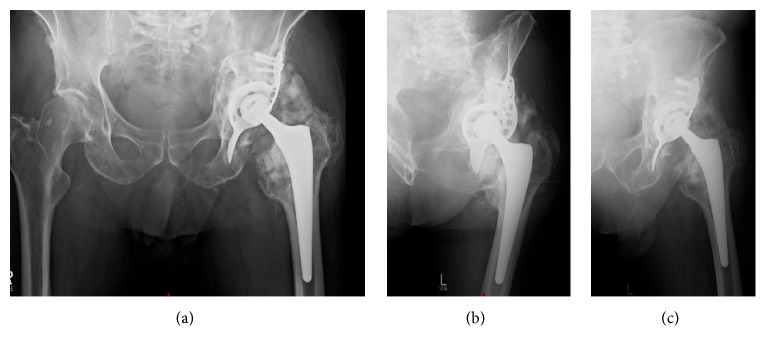
Follow-up X-ray (a.p., obturator and ala-view) one year postoperatively.

The X-rays at follow-up demonstrated correct position and articulation of the revision THA with no signs of loosening of the Burch-Schneider cage. The bone graft substitute had remodelled to a great extent into trabecular bone (Figure 4). However, heterotopic ossifications (HO) had formed around the hip joint (Arcq classification grade II, Booker classification grade III) [8, 9].

### 2.2. Follow-Up at Two Years and Eight Months

**Figure 5:**
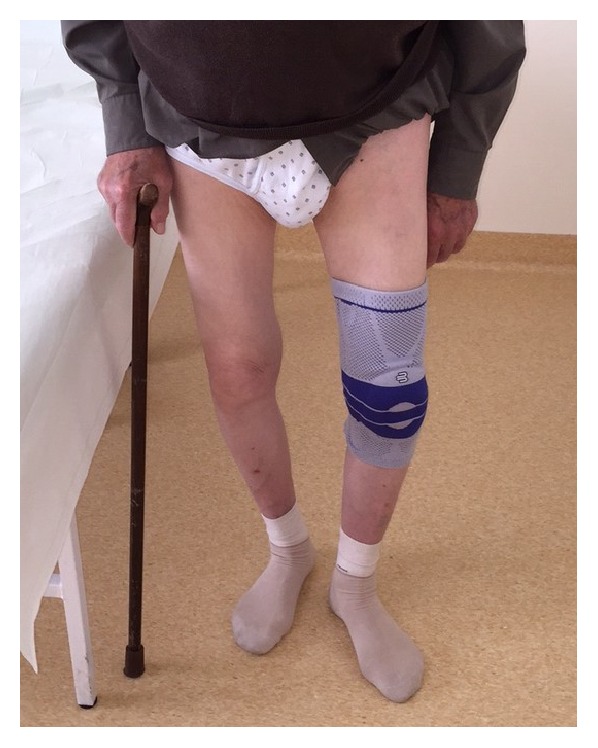
Two years and eight months after surgery. Patient is ambulatory using a walking stick.

At the 2-year follow-up examination the patient was ambulatory using a walking stick (Figure 5). He was independent with self-care in his house and had no pain during standing and sitting. His gait was slow and limping with minor pain in the left hip.

**Figure 6:**
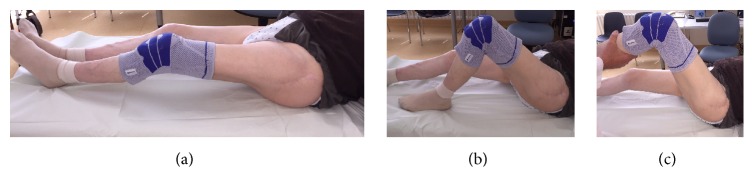
Clinical examination two years and eight months postoperatively. Extension/flexion of the left hip 0°-20°-70°, abduction/adduction 40°-0°-10°, and rotation 10°-0°-10°.

Extension/flexion of the left hip was 0°-20°-70°, abduction/adduction 40°-0°-10°, and external/internal rotation 10°-0°-10° (Figure 6). The patient was satisfied with the result of the revision surgery and in general emotionally well.

**Figure 7:**
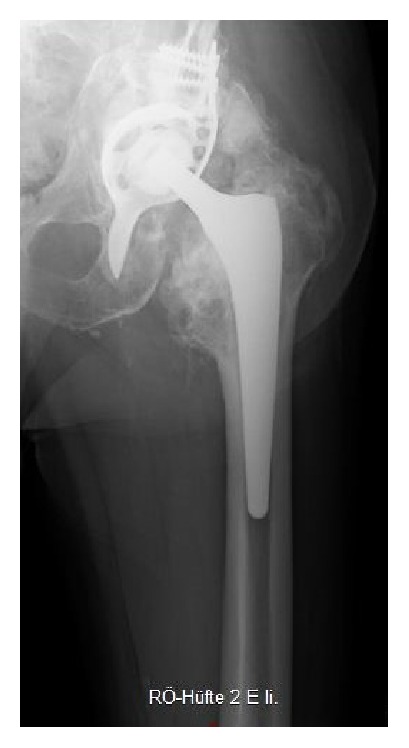
Follow-up X-ray of left hip a.p. two years and eight months postoperatively.

Radiographically, complete remodelling of CERAMENT|BONE VOID FILLER is now displayed (Figure 7). No signs of loosening of the antiprotrusio cage or movement of the revision THA were found.

The HO had increased; the joint seemed to be completely encased (Arcq classification grade II, Booker classification grade III) [8, 9].

## 3. Discussion

Only a few cases of periprosthetic acetabular fractures have been reported [2, 7, 10]. Two types of periacetabular fractures should be distinguished: (I) with well-fixed cup-component and (II) with loosening of the THA cup-component. In situation one, a conservative approach is usually recommended [7]. However, to ensure early mobilisation and reduce cup loosening at follow-up, Gras et al. recently advocated a navigated percutaneous screw fixation technique of periprosthetic acetabular fractures [11].

A preoperative CT scan would have been beneficial in our case to describe the fracture and to exclude a pelvic discontinuity, which would have needed additional fixation of the posterior acetabular column.

**Figure 8:**
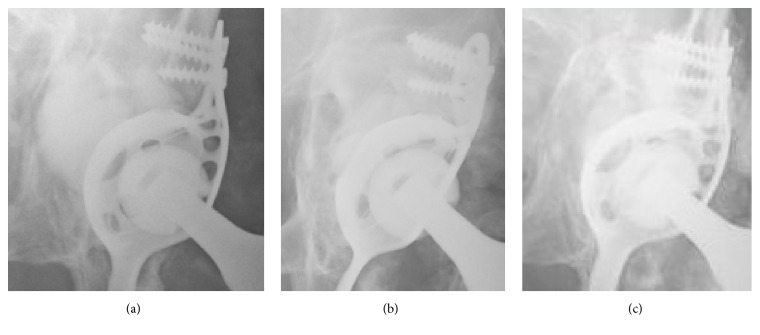
Enlargement of the a.p. X-rays of the left hip: at (a) two days, (b) one year, and (c) two years and eight months postoperatively: continuing remodelling of CERAMENT|BONE VOID FILLER into bone.

Risk factors for a periprosthetic acetabular fracture are age, osteoporosis, female gender, overreaming of the acetabulum, and use of a cementless cup. In our case, we were faced with a type two fracture with loosening of the cup-component. In a revision surgery all components of the THA were removed, the acetabular fracture was reduced, and an antiprotrusio cage, a cemented cup, and an uncemented stem were implanted. This approach has been described before, combined with cancellous allograft to treat the acetabular bone loss [12]. We used the injectable calcium sulphate/hydroxyapatite bone graft substitute CERAMENT|BONE VOID FILLER to reconstruct the bony defect. This material had remodelled into bone to a great extent at the X-ray follow-up after one year (Figure 8). The remodelling was almost complete after two years and eight months. Other bone graft substitutes tend to be resorbed too early (e.g., calcium sulphate) [13] to allow bone regeneration or do not remodel into bone after several years (e.g., calcium phosphate) [14]. The combination of 60% calcium sulphate and 40% hydroxyapatite seems to fulfil the criterion of equal resorption and new bone formation at the same pace [15]. The advantages of remodelling are the prevention of foreign body reaction or infection and induction of new bone stock, which may be required in further hip revision surgery.

In the reported case, the range of motion of the left hip is significantly reduced by HO, which has developed despite the prophylactic use of Diclofenac. HO has been reported in about 10% of primary THA [16], but usually without any limitation of the range of motion. The genesis of HO is still not completely understood [17]. Risk factors are joint arthroplasty, spinal cord injury, traumatic brain injury, blast trauma, elbow and acetabular fractures, and thermal injury [17]. In addition to drug prophylaxis, usually with Indomethacin, prophylactic radiation can be used to prevent HTO. The bone graft substitute used is osteoconductive, but in contrast to growth factors not osteoinductive. An increased rate of HO has not been previously reported in the use of this bone graft substitute [18, 19] and is the subject of a current case series in acetabular revision surgery.
